# Associations of cardiometabolic polygenic risk scores with cardiovascular disease in African Americans

**DOI:** 10.21203/rs.3.rs-3228815/v1

**Published:** 2023-08-30

**Authors:** Workalemahu Tsegaselassie, Ying Jian, Gebremeskel G. Berhanu, Lu Tianyuan, Mohanty April, Elfassy Tali, Tekola-Ayele Fasil, Thornton A. Timothy, Cohen Jordana, Irvin R. Marguerite, Silver M. Robert, Varner W. Michael, Yaffe Kristine, Fornage Myriam, Lloyd-Jones M. Donald, Sims Mario, Shimbo Daichi, Yano Yuichiro, Muntner Paul, Bress Adam

**Affiliations:** University of Utah Health; University of Utah; University of Michigan; Lady Davis Institute for Medical Research, Jewish General Hospital; University of Utah; University of Miami; National Institutes of Health; Regeneron Genetics Center; University of Pennsylvania; University of Alabama at Birmingham; University of Utah Health; University of Utah Health; University of California San Francisco; University of Texas Health Science Center at Houston; Northeastern University; University of Mississippi Medical Center; Columbia University Irving Medical Center; Duke University; University of Alabama at Birmingham; University of Utah

**Keywords:** Cardiovascular disease, polygenic risk score, blood pressure, lipids, cardiometabolic, African Americans

## Abstract

**Background:**

Cardiovascular disease (CVD) is a complex disease, and genetic factors contribute individually or cumulatively to CVD risk. While African American women and men are disproportionately affected by CVD, their lack of representation in genomic investigations may widen disparities in health. We investigated the associations of cardiometabolic polygenic risk scores (PRSs) with CVD risk in African Americans.

**Methods:**

We used the Jackson Heart Study, a prospective cohort study of CVD in African American adults and the predicted atherosclerotic cardiovascular disease (ASCVD) 10-year risk. We included 40–79 years old adults without a history of coronary heart disease (CHD) or stroke at baseline. We derived genome-wide PRSs for systolic blood pressure (SBP), diastolic blood pressure (DBP), total cholesterol, LDL cholesterol, hemoglobin A1c (HbA1c), triglycerides, and C-reactive protein (CRP) separately for each of the participants, using African-origin UK Biobank participants’ genome-wide association summary statistics. We estimated the associations between PRSs and 10-year predicted ASCVD risk adjusting for age, sex, study visit date, and genetic ancestry using linear and logistic regression models.

**Results:**

Participants (n=2,077) were 63% female and 66% never-smokers. They had mean (SD) 56 (10) years of age, 127.8 (16.3) mmHg SBP, 76.3 (8.7) mmHg DBP, 200.4 (40.2) mg/dL total cholesterol, 51.7 (14.7) mg/dL HDL cholesterol, 127.2 (36.7) mg/dL LDL cholesterol, 6.0 (1.3) mmol/mol HbA1c, 108.9 (81.7) mg/dL triglycerides and 0.53 (1.1) CRP. Their median (interquartile range) predicted 10-year predicted ASCVD risk was 8.0 (4.0–15.0). Participants in the >75^th^ percentile for HbA1c PRS had 1.42 percentage-point greater predicted 10-year ASCVD risk (1.42 [95% CI: 0.58–2.26]) and higher odds of ≥10% predicted 10-year ASCVD risk (OR: 1.46 [95% CI: 1.03–2.07]) compared with those in the <25^th^ percentile for HbA1c PRS. Participants in the >75^th^ percentile for SBP PRS had higher odds of ≥10% predicted 10-year ASCVD risk (OR: 1.52 [95% CI: 1.07–2.15]) compared with those in the <25^th^ percentile for SBP PRS.

**Conclusion:**

Among 40–79 years old African Americans without CHD and stroke, higher PRSs for HbA1c and SBP were associated with CVD risk. PRSs may help stratify individuals based on their clinical risk factors for CVD early prevention and clinical management.

## Introduction

Cardiovascular disease (CVD), which includes coronary heart disease (CHD), sudden cardiac death/sudden cardiac arrest, stroke/transient ischemic attack, and peripheral arterial disease, is a complex multifactorial disease.[1] Genetic factors contribute individually or cumulatively to CVD risk.[2–5] CVD disproportionately affects African American women and men,[1, 6] and their lack of representation in genomic investigations may widen disparities in cardiovascular health. While non-genetic risk factors for CVD predict clinical outcomes equally well in African Americans and others,[1, 7, 8] risk prediction efforts that incorporate polygenic risk scores (PRSs) tailored towards African Americans are limited but may reduce disparities in guiding disease prevention efforts.[9–13] In the context of risk prediction and prevention of complex multifactorial diseases such as CVD, current guidelines suggest population-specific genetic investigations to reduce disparities.[1, 11, 12]

The availability of the UK biobank genome-wide association study (GWAS) summary statistics provided the opportunity to derive racial/ethnic-specific PRSs to estimate part of the individual risk for common traits and CVD risk factors (including blood pressure, lipids and inflammatory markers).[14, 15] Identifying individuals with the earliest indication of a predisposition to vascular disease (atherosclerosis), including genetic predisposition, may allow early preventative action to be taken in high-risk individuals.[13, 16, 17] A recent study has shown that systolic blood pressure (SBP) and diastolic blood pressure (DBP) PRSs are highly predictive of CVD risk in a multi-ethnic cohort.[18] However, attenuated effects of genetic risk on cardiometabolic traits in African Americans[18–21] and the limited portability of PRSs across populations warrant further investigation using GWASs from African-ancestry individuals.[7, 9] Furthermore, the associations of PRSs based on other CVD risk factors such as lipid and inflammatory markers with CVD risk in African Americans are unknown. Therefore, we investigated the associations of SBP, DBP, hemoglobin A1c, cholesterol, triglycerides and C-reactive protein PRSs, separately, with CVD risk in African Americans.

## Methods

### Study design and population

The Jackson Heart Study (JHS) is a prospective cohort study designed to evaluate CVD risk among African American participants between 2000–2004 (Exam 1), 2005–2008 (Exam 2), 2005–2007 (Exam 4) and 2010–2011 (Exam 5). Briefly, the JHS enrolled a representative sample of ≥ 20 years old African American participants from the Atherosclerosis Risk in the Community site in the urban and rural Jackson-Mississippi metropolitan tri-county area (Hinds, Madison, and Rankin counties).[22–25] The institutional review board (IRB) governing human subjects’ research at the participating institutions approved the JHS protocol and all data collection procedures. All research was performed in accordance with relevant guidelines/regulations and the study participants provided written informed consent. The current analysis was approved by the IRB of the University of Alabama at Birmingham.

### Phenotype ascertainment

The primary outcome of this study was 10-year predicted atherosclerotic cardiovascular disease (ASCVD) risk defined using the Pooled Cohort Equations to estimate.[26–28] Briefly, the Pooled Cohort Equations estimator predicted the race-sex specific 10-year ASCVD risk using individuals’ age, total cholesterol, high-density lipoprotein cholesterol, clinical systolic blood pressure, and antihypertensive medication use, current smoking, and diabetes mellitus statuses. Estimated 10-year predicted ASCVD risk is used to guide treatment decision for statins and antihypertensive medications for primary prevention. As such, we used the 10-year predicted ASCVD risk also because it serves as a composite for incident coronary heart disease and stroke, which are major adverse CVD events.[27]

The 10-year predicted ASCVD risk was estimated among 40–79 years old adults without CVD and history of stroke at baseline (Exam 1). Therefore, among a total of 3,029 JHS participants, we excluded participants < 40 years of age or > 79 years of age (n = 420) and participants with history of CHD (n = 228) or stroke (n = 94). The final analysis included 2,077 participants with non-missing ASCVD record.

Baseline (Exam 1) systolic blood pressure (SBP) and diastolic blood pressure (DBP) were each measured in mmHg units by taking the average of two readings using Hawksley random-zero sphygmomanometers in rested and seated participants. Plasma high-density lipoprotein (HDL), total cholesterol and triglycerides were measured using standard enzymatic methods, hemoglobin A1c (HbA1c) was measured using high-performance liquid chromatography and C-reactive protein (CRP, mg/dL) was measured by immunoturbidimetric CRP-latex assay in mg/dL units, using previously described methods.[29, 30] [31] We evaluated LDL cholesterol and total cholesterol mainly due to the confirmed causal associations between LDL cholesterol and non-HDL cholesterol with coronary events in Mendelian randomization studies.[32]

### Genotyping, imputation and quality control

JHS participants’ single-nucleotide polymorphism (SNP) genotyping was performed using the Affymetrix 6.0 SNP Array (Affymetrix, Santa Clara, Calif). SNPs with minor allele frequency (MAF) ≥ 1%, a call rate ≥ 90%, and a Hardy-Weinberg Equilibrium (HWE) P-value > 10^−6^ were used for imputation to the 1000 Genomes reference panel.[33] The first 10 principal components were estimated to represent global ancestry based on linkage-disequilibrium pruned set of SNPs with MAF > 0.05.[34] Outliers based on the principal component scores for global ancestry, sample swaps, duplicates, and one of each pair of monozygotic twins were excluded. Participants with a mismatch between pedigree vs genetic sex were also removed.[33] Out of N = 47,101,766 imputed SNPs in the JHS, the present analysis included N = 9,360,683 SNPs with MAF > 0.05%, genotype call rate ≥ 90% and HWE P-value > 10^−6^.

### Polygenic risk score derivation

We derived genome-wide PRSs for each trait (SBP, DBP, HDL and total cholesterol, HbA1c, triglycerides and CRP) using the United Kingdom (UK) Biobank (UKBB) genome-wide association summary statistics data from GWASs conducted in African-origin study participants. The UKBB is independent of the JHS and is based on a prospective population-based study of 40-to 69-years old participants recruited across 22 assessment centers throughout the UK from 2006–2010.[14, 15] Genotyping was performed on either the Affymetrix Axiom array or the UK BiLEVE Axiom array.[14] Quality control steps removed individuals with a genotype call rate < 98%, SNPs with a call rate of < 98%, SNPs with a MAF < 1% or those which deviated from Hardy–Weinberg equilibrium (p < 5 × 10 − 6). The summary statistics data (publicly available at: https://pan.ukbb.broadinstitute.org) included effect estimates (Beta), P-value, and risk/reference alleles for genome-wide single-nucleotide polymorphisms (SNPs) with minor-allele-frequency ≥ 1%. The genomic inflation factor for GWASs of each trait was around 1.00, suggesting no inflation in the summary statistics (Table 3). The PRSs were derived by applying clumping (to prune redundant correlated effects caused by LD between SNPs) and thresholding (to derive SNP effects at different P-value thresholds), a method implemented in PRSice2.[35] [36] We applied the clumping and thresholding method rather than the more recent LDpred[37] or lassosum[38] methods that may still need further study using data sources with diverse study participants.[13] Briefly, we used an r^2^ ≥ 0.2 and 250-kilo base window for clumping. Each PRS was derived by summing the blood pressure-, lipids-, and inflammatory marker-increasing alleles (i.e., Beta estimates), separately, across genome-wide SNPs with P-value thresholds between 0 and 0.99 at increments of 5e^−5^ using a “high resolution scoring” approach that provided the most predictive PRS for 10-year predicted ASCVD risk. The Beta estimates from the UKBB summary data were applied to PRSs as weights. PRSs with the greatest number of SNPs included in the model and explaining the most variance in the traits were selected for downstream analyses.

### Statistical analysis

The characteristics of our study participants were summarized by 10-year predicted ASCVD risk groups using mean ± standard deviation or median and interquartile ranges for continuous variables, and counts (frequency) for categorical variables. The correlations of measured blood pressure, lipid and inflammatory markers with the respective PRSs were examined using the Pearson correlation. To estimate 10-year ASCVD absolute risk differences comparing individuals grouped by PRS tertiles, linear regression models were used. To determine the associations of PRS tertiles with high vs. low, defined as ≥ 10% vs. <10% 10-year predicted ASCVD risk, logistic regression models were used. This approach aligns with the 2017 hypertension guideline from the American College of Cardiology/American Heart Association (ACC/AHA), which recommends stratifying individuals by ≥ 10% vs. <10% 10-year predicted ASCVD risk.[39] The models were adjusted for age, sex, study visit date, global genetic ancestry, and sex-by-age interaction term. The tests for each PRSs were considered independent tests because different genetic factors and mechanisms may contribute differently towards the CVD risks associated with blood pressure, lipids and inflammatory factors. Analyses were conducted using R, PLINK 1.9, PRSice-2 2.1.3.beta,[35, 40] and SAS version 9.4 (SAS Institute Inc).

## Results

Among the total JHS participants (n = 2,077), 63% were female and 66% were never-smokers (Table 1). Participants had mean (SD) 56 (10) years of age, 127.8 (16.3) mmHg SBP, 76.3 (8.7) mmHg DBP, 200.4 (40.2) mg/dL total cholesterol, 51.7 (14.7) mg/dL HDL cholesterol, 127.2 (36.7) mg/dL LDL cholesterol, 6.0 (1.3) mmol/mol HbA1c, 108.9 (81.7) mg/dL triglycerides and 0.53 (1.1) CRP. Their median (interquartile range) percentage-point 10-year predicted ASCVD risk was 8.0% (4.0%–15.0%). In comparison to participants with < 10% 10-year predicted ASCVD risk (n = 1,179), there were higher proportions of males (46% vs. 30%) and smokers (41% vs 27%) among participants with ≥ 10% 10-year predicted ASCVD risk (n = 898). Furthermore, participants with ≥ 10% 10-year predicted ASCVD risk had higher mean (SD) (64 [8] vs. 50 [7]), 135.6 (16.3) vs. 121.6 (13.1) mmHg SBP, 76.6 (8.8) vs. 76.3 (8.5) mmHg DBP, 205.8 (42.4) vs. 196.4 (38.1) mg/dL total cholesterol, 130.9 (37.0) vs. 124.6 (36.3) mg/dL LDL cholesterol, 6.4 (1.5) vs. 5.6 (0.8) mmol/mol HbA1c, 127.6 (104.7) vs. 94.5 (53.8) mg/dL triglycerides and 0.54 (1.4) vs. 0.53 (0.8) CRP, and lower 50.1 (14.7) vs 53.0 (14.5) mg/dL HDL cholesterol, in comparison to those with ≥ 10% 10-year predicted ASCVD risk.

HbA1c and SBP PRSs were significantly associated with higher 10-year predicted ASCVD risk (Table 2). Specifically, Participants in the > 75th percentile for HbA1c PRS had a 1.42 percentage-point higher 10-year predicted ASCVD risk (95% CI: 0.58–2.26) and higher odds of ≥ 10% 10-year predicted ASCVD risk (OR: 1.46 [95% CI: 1.03–2.07]) compared with those in the < 25th percentile for HbA1c PRS. Participants in the > 75th PRS percentile for SBP had increased odds of ≥ 10% 10-year predicted ASCVD risk (OR = 1.52; 95% CI: 1.07–2.15) and increased but not statistically significant percentage-point in 10-year predicted ASCVD risk (0.82; 95% CI: −0.01–1.70) compared with those in the < 25th PRS percentile for SBP.

DBP, LDL cholesterol, triglycerides and CRP PRSs were associated with increased but not statistically significant 10-year predicted ASCVD risk. Specifically, while participants in the > 75th PRS percentile for DBP had lower percentage-point in 10-year predicted ASCVD risk (−0.60 95% CI: −1.43–0.22), they had higher odds of ≥ 10% 10-year predicted ASCVD risk (OR = 1.20; 95% CI: 0.84–1.70), compared with those in the < 25th PRS percentile for DBP. In addition, participants in the > 75th PRS percentile for LDL cholesterol had higher percentage-point in 10-year predicted ASCVD risk (0.56 95% CI: −0.27–1.38) and higher odds of ≥ 10% 10-year predicted ASCVD risk (OR = 1.07; 95% CI: 0.76–1.52), compared with those in the < 25th PRS percentile for LDL cholesterol. Similarly, participants in the > 75th percentile for triglycerides had higher percentage-point 10-year predicted ASCVD risk (0.83; 95% CI: 0.0–1.70) and higher odds of ≥ 10% 10-year predicted ASCVD risk (OR = 1.02; 95% CI: 0.72–1.45) in comparison to the reference group. Lastly, participants in the > 75th percentile for CRP had higher percentage-point 10-year predicted ASCVD risk (0.32; 95% CI: −0.51–1.16) and higher odds of ≥ 10% 10-year predicted ASCVD risk (OR = 1.11; 95% CI: 0.78–1.58) in comparison to the reference group.

**Figure 1:**
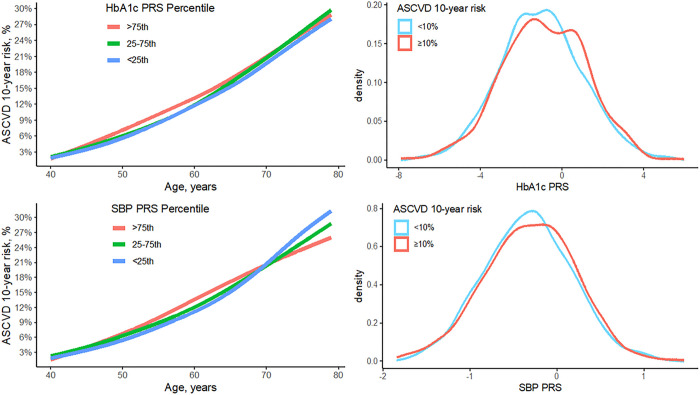
Distributions of HbA1c and SBP PRSs by absolute and high/low 10-year predicted ASCVD risk

Total cholesterol PRS was excluded from further analysis due to limited number of SNPs (n = 2) predictive of the phenotype in the JHS (Table 3). The number of SNPs included in PRS prediction model for SBP, DBP, LDL cholesterol, HbA1c, triglycerides and CRP ranged from 136 to 183,104. The summary of the HbA1c and SBP PRSs and their distribution by 10-year predicted ASCVD risk display individuals in the higher PRS percentiles deferred by their 10-year predicted ASCVD absolute and relative risks (Fig. 1). The summary of the DBP, LDL cholesterol, triglycerides and CRP PRSs and their distribution by 10-year predicted ASCVD risk is shown in **Supplementary Fig. 1.**

## Discussion

Among 40–79 year old African Americans without baseline CHD and stroke, those in the highest HbA1c PRS tertile had higher percentage-point in 10-year predicted ASCVD risk and higher odds of ≥ 10% 10-year predicted ASCVD risk compared with those in the lowest HbA1c PRS tertile. In addition, individuals in the highest SBP PRS tertile had higher odds of ≥ 10% 10-year predicted ASCVD risk compared with those in the lowest SBP PRS tertile. Correspondingly, individuals in the highest DBP, LDL cholesterol, triglycerides and CRP PRS tertiles had higher but not statistically significant 10-year predicted ASCVD risks compared with those in lowest PRS tertiles. The associations of HbA1c and SBP PRSs with 10-year predicted ASCVD risk were independent of participants’ age, sex, study visit date and genetic ancestry, suggesting utility of PRSs for capturing long-term exposure to a heritable CVD risk.

Our study evaluated PRSs estimated in a large African-ancestry GWAS within an independent but limited sample size of African-Americans. The association between higher SBP PRS with incident CVD was recently reported and validated in a large pooled data that included participants from multiple ancestries.[18] Several prior studies have also reported associations of blood pressure PRSs with CVD risk,[41–44] but even in pan-ancestry GWASs, European-ancestry groups remain over-represented. In the present study, we also observed that HbA1c and SBP PRSs derived from European-ancestry UKBB data were associated with 10-year predicted ASCVD risk in the JHS, owing to the large sample of the UKBB GWAS. Though pan-ancestry data sources have provided statistical power to evaluate PRSs while providing the potential to address transferability of polygenic risk prediction across different ancestries,[45] several challenges have also been underscored.

First, the overestimation of CVD risk in non-European ancestries were noted when applying prediction models derived from pooled cohorts over-represented by a specific-ancestry.[7, 46] Due to allele frequency differences,[9] hypertension PRS distributions, for example, differed by proportion of specific genetic ancestry.[13] While African genetic ancestry of African Americans ranges from 30–100%, genetic heterogeneity by admixture may influence the sums of alleles, which are used to construct PRSs. Second, when PRS distributions differ across race/ethnicities, PRSs based on pan-ancestry GWAS data pose a challenge on predicting individuals “at risk” for cardiometabolic factors and CVD.[13, 47] For example, individuals may be misclassified as “at risk” when admixed individuals are not accurately represented by any specific PRS distribution. Alternative strategies to overcome the challenge include CVD risk prediction tailored towards specific groups, such as African American women and men, [1, 6] who are disproportionately affected by CVD.[9–13] Current guidelines also suggest population-specific genetic investigations to reduce disparities and guide disease prevention efforts in the context of multi-factorial complex diseases like CVD.[1]

Third, studies with flexible risk prediction models that account for non-genetic risk factors and their interactions have been limited but are critical to reducing disparities and guiding disease prevention efforts.[9–13] For example, a recent study highlighted candidate SNPs that modified the association between perceived discrimination and elevated SBP in the JHS.[48] In the present study, HbA1c and SBP PRSs were associated with 10-year predicted ASCVD risk after additionally accounting for smoking and insurance statuses (data not shown), however, the associations were not modified by smoking or insurance statuses. Socio-economic stressors that disproportionately affect population groups are relevant and larger studies that evaluate PRS-by-environment interactions,[13, 48] while taking careful considerations with respect to how the sample populations’ genetic ancestry compares to the that of the training data are needed.[12]

Furthermore, the role that polygenic risks for elevated lipid and inflammatory factors, as cardiometabolic markers of plaque stability, play on CVD risk among African-ancestry individuals is unknown. Evaluating lipid and inflammatory marker PRSs in CVD risk may address risk misclassification due to genetic influences on the performances of their assays and allow investigation into pleiotropy between the traits. For example, genetic factors of hemoglobin influenced the performance of some HbA1c assays, potentially leading to misclassification of individuals who have achieved glucose control and may be at lower risk for type-2 diabetes.[10, 49, 50] Therefore, the lack of knowledge regarding genetic variants affecting HbA1c measurement independently of blood glucose concentration may exacerbate health disparities due to misdiagnosis and treatment inaccuracy.[51]

The associations of HbA1c genetic variants with type-2 diabetes are known,[51, 52] including in a recent study that demonstrated transferability of the findings in individuals that share African-ancestry.[10] However, a 1%-unit increase in HbA1c was also associated with a 20–50% increased CVD risk in individuals without type-2 diabetes. [53] The prevalence of type-2 diabetes in the JHS participants included in this study is 24%, which is higher than the national type-2 diabetes prevalence in African Americans, but HbA1c levels may also contribute to CVD risk in diabetic individuals. HbA1c increases dyslipidemia, hypertension, CRP, oxidative stress and blood viscosity leading to CVD.[54] Among European-ancestry individuals, a mendelian randomization study has shown that known HbA1c SNPs are associated with CVD risk.[55]

In European-ancestry individuals, a Mendelian randomization study suggested a causal association between cholesterol SNPs and type-2 diabetes.[56] Triglyceride is not directly atherogenic but represents an important biomarker of CVD risk because of its association with atherogenic remnant particles.[57] Other inflammatory markers like CRP can induce inflammatory changes in endothelial and smooth muscle cells and are related to CVD risk.[58] Although we were unable to assess the genetic overlap between HbA1c, cholesterol, triglycerides in our study, methods appropriate in leveraging pleiotropy in larger studies have been developed.[59, 60] These methods may enhance both discovery and genetic associations while creating potentially more powerful PRS for each of the traits.[13]

Lastly, our study has several other limitations and strengths. The sample size for African-origin UKBB participants ranged from 5,290–6,551, representing a small sample size for detecting genome-wide significant common variants. However, the UKBB is the largest publicly available GWAS summary data with representative samples across various ancestries. Furthermore, our study excluded < 40 years old individuals because 10-year predicted ASCVD risk, a composite outcome of CHD and stroke, which is the focus of most primary prevention guidelines, was estimated in 40–79 years old adults.[26–28] However, PRSs may be more predictive of CVD risk in younger asymptomatic individuals than individuals with symptomatic CVD risk profiles.[5] While plaque build-up in the vasculature can begin during early adulthood, initially asymptomatically, progressing thereafter,[61, 62] identifying the earliest indication of a predisposition to such build-up, including genetic predisposition, may allow early preventative action to be taken in high-risk individuals. Furthermore, in the present study, we found associations limited to HbA1c and SBP PRSs with CVD risk but not with other cardiometabolic trait PRSs. Modest SNP effects on DBP levels have been previously reported.[18, 63]

In the present study, we derived PRSs using summary data that may be tailored towards African-ancestry individuals, i.e., target populations where there may be negligible differences in LD or causal allele frequencies with African-origin UK populations.[9] However, as underlying genetic ancestry may influence the established genetic associations with complex diseases, local genetic ancestry (characterized as ancestral states at each genetic locus) estimates should be included in regression models. In addition, larger base, target and validation GWASs in African-ancestry data are required to increase predictive power of PRSs in African-ancestry individuals. Lastly, in individuals with low polygenic risk, rare variant screening may enhance risk prediction for common diseases and guide appropriate risk stratification.[64]

## Conclusions

Among 40–79 years old African Americans without baseline CHD and stroke, our study showed that higher PRSs for SBP and HbA1c are associated with increased probability of developing CVD. CVD risk prediction using PRSs may help stratify individuals based on their clinical risk factors and by their polygenic scores for CVD early prevention and clinical management. The CVD risk prediction efforts should also incorporate detailed assessment of the population to be investigated and the consequent prevention and clinical management strategies that would then be offered.

## Figures and Tables

**Table 1:** Baseline characteristics of the JHS participants with genome-wide association data

Characteristics	10-year predicted ASCVD risk		Total (n = 2077)
	≥10% (n = 898)	<10% (n = 1179)	
Age (years), mean (SD)	63.9 (8.0)	50.4 (7.4)	56.4 (10.2)
Sex, n (%)	-	-	-
Female	486 (54.1)	821 (69.6)	1437 (62.8)
Male	412 (45.9)	358 (30.4)	850 (37.2)
Smoking, n (%)	-	-	-
Never	526 (58.6)	867 (73.5)	1516 (66.4)
Ever	372 (41.4)	312 (26.5)	767 (33.6)
Body-mass-index (kg/m^2^), mean (SD)	-	-	-
Normal, n (%)	106 (11.8)	157 (13.3)	285 (12.5)
Overweight, n (%)	299 (33.4)	391 (33.2)	758 (33.2)
Obese, n (%)	486 (54.2)	629 (53.4)	1233 (54.0)
Insurance, n (%)	-	-	-
Private	515 (57.4)	928 (78.7)	1560 (68.2)
Medicare/Medicaid	272 (30.3)	71 (6.0)	404 (17.7)
**Cardiometabolic measurements, mean (SD)**	-	-	-
Systolic Blood Pressure (mmHg)	135.6 (16.3)	121.6 (13.1)	127.8 (16.3)
Diastolic Blood Pressure (mmHg)	76.6 (8.8)	76.3 (8.5)	76.3 (8.7)
LDL Cholesterol (mg/dL)	130.9 (37.0)	124.6 (36.3)	127.2 (36.7)
HDL Cholesterol (mg/dL)	50.1 (14.7)	53.0 (14.5)	51.7 (14.7)
HbA1c (mmol/mol)	6.4 (1.5)	5.6 (0.8)	6.0 (1.3)
Triglycerides (mg/dL)	127.6 (104.7)	94.5 (53.8)	108.9 (81.7)
Total Cholesterol (mg/dL)	205.8 (42.4)	196.4 (38.1)	200.4 (40.2)
eGFR (mL/min/1.73 m2)	86.8 (20.1)	99.7 (17.5)	93.6 (20.5)
Albumin-to-creatinine ratio	0.07 (0.33)	0.02 (0.11)	0.04 (0.2)
C-reactive protein	0.54 (1.4)	0.53 (0.8)	0.53 (1.1)
**Medication use, n (%)**	-	-	-
Antihypertension	673 (74.9)	421 (35.7)	1229 (54.1)
Diabetes	226 (25.2)	42 (3.6)	371 (16.4)
Statin	176 (19.6)	75 (6.4)	284 (12.5)
Beta blocker	114 (13.6)	75 (6.7)	212 (10.0)
Calcium channel blocker	247 (29.5)	139 (12.4)	440 (20.6)
Antiarrhythmic	42 (4.7)	25 (2.2)	73 (3.2)
**Outcome events**	-	-	-
Prevalent Hypertension, n (%)	732 (81.5)	476 (40.4)	1349 (59.0)
Incident Hypertension, n (%)	101 (11.3)	322 (27.3)	460 (20.1)
Time-to-incident hypertension (days), mean, (min-max)	252.7 (0–3417.0)	852.0 (0–4283.0)	536.4 (0.0–4283.0)
Incident coronary heart disease, n (%)	12 (1.4)	4 (0.3)	23 (1.0)
Prevalent diabetes mellitus, n (%)	356 (39.6)	81 (6.9)	552 (24.2)
**CVD/CHD 10-Year Risk, median (IQR)**	-	-	-
American College of Cardiology - American Heart Association - Atherosclerotic Cardiovascular Disease (ASCVD)	15.5% (13.0% – 23.0%)	4.0% (2.0% – 6.0%)	8.0% (4.0% – 15.0%)
Framingham Risk Score-Cardiovascular Disease (FRS CVD)	18.0% (14.0% – 25.0%)	5.0% (3.0% – 10.0%)	11.0% (5.0% – 19.0%)
Framingham Risk Score-Coronary Heart Disease (FRS CHD)	15.0% (9.0% – 22.0%)	6.0% (3.0% – 9.0%)	8.0% (5.0% – 14.0%)
Framingham Risk Score- Adult Treatment Panel (III) – Coronary Heart Disease (FRS ATP CHD)	8.0% (5.0% – 16.0%)	1.0% (1.0% – 4.0%)	4.0% (1.0% – 8.0%)
**Systolic Blood Pressure PRS, n (%)**	-	-	-
< 25th percentile	225 (25.1)	310 (26.3)	586 (25.6)
25th – 75th percentile	443 (49.3)	587 (49.8)	1140 (49.9)
>75th percentile	230 (25.6)	282 (23.9)	561 (24.5)
**Diastolic Blood Pressure PRS, n (%)**			
< 25th percentile	211 (23.5)	289 (24.5)	558 (24.4)
25th – 75th percentile	456 (50.8)	584 (49.5)	1133 (49.5)
>75th percentile	231 (25.7)	306 (26.0)	596 (26.1)
**LDL Cholesterol PRS, n (%)**	-	-	-
< 25th percentile	225 (25.1)	304 (25.8)	573 (25.1)
25th – 75th percentile	430 (47.9)	602 (51.1)	1148 (50.2)
>75th percentile	243 (27.1)	273 (23.2)	566 (24.8)
**HbA1c PRS, n (%)**	-	-	-
< 25th percentile	206 (22.9)	290 (24.6)	560 (24.5)
25th – 75th percentile	431 (48.0)	619 (52.5)	1147 (50.2)
>75th percentile	261 (29.1)	270 (22.9)	580 (25.4)
**Triglycerides PRS, n (%)**	-	-	-
< 25th percentile	218 (24.3)	308 (26.1)	581 (25.4)
25th – 75th percentile	453 (50.4)	580 (49.2)	1137 (49.7)
>75th percentile	227 (25.3)	291 (24.7)	569 (24.9)
**C-reactive protein PRS, n (%)**	-	-	-
< 25th percentile	217 (24.2)	284 (24.1)	558 (24.4)
25th – 75th percentile	457 (51.0)	598 (50.7)	1160 (50.7)
>75th percentile	224 (24.9)	297 (25.2)	569 (24.9)

**Table 2:** Associations of cardiometabolic marker PRSs with 10-year predicted ASCVD risk

PRS Percentiles	10-year predicted ASCVD risk^1^	
	Absolute (%)^2^	≥ 10% vs. <10%^3^
SBP		
< 25th (ref)	-	-
25–75th	0.33 (−0.39–1.1)	1.21 (0.89–1.65)
>75th	0.82 (−0.01–1.7)	1.52 (1.07–2.15)
DBP		
< 25th (ref)	-	-
25–75th	−0.40 (−1.11–0.33)	1.14 (0.84–1.54)
>75th	−0.60 (−1.43–0.22)	1.20 (0.84–1.70)
LDL Cholesterol		
< 25th (ref)	-	-
25–75th	−0.25 (−0.96–0.46)	0.97 (0.72–1.31)
>75th	0.56 (−0.27–1.38)	1.07 (0.76–1.52)
HbA1c		
< 25th (ref)	-	-
25–75th	0.53 (−0.19–1.26)	1.02 (0.75–1.38)
>75th	1.42 (0.58–2.26)	1.46 (1.03–2.07)
Triglycerides		
< 25th (ref)	-	-
25–75th	0.69 (−0.02–1.40)	1.24 (0.92–1.68)
>75th	0.83 (0.0–1.7)	1.02 (0.72–1.45)
CRP		
< 25th (ref)	-	-
25–75th	0.15 (−0.57–0.87)	1.06 (0.78–1.44)
> 75th	0.32 (−0.51–1.16)	1.11 (0.78–1.58)

1 Estimates are effect size (95% confidence interval) adjusted for age, age^2^, sex, study visit, genetic ancestry (10 principal components) and the interaction term for age and sex

2 10-year predicted ASCVD absolute risk

3 10-year predicted ASCVD risk using the ACC/AHA 2017 hypertension guideline to stratify individuals by ≥ 10% vs. <10% 10-year predicted ASCVD risk

**Table 3:** Summary of UKBB data and PRSs in JHS

	UK Biobank GWAS			PRS in JHS				
	Phenotype code	Number of Participants	λ (Genomic control)	Number of SNPs included	mean (SD)	Interquartile Range	R^2^	P-value 2
**SBP**	4080	6551	1.00	183104	−0.32 (0.51)	0.69	0.045	0.01
**DBP**	4079	6551	1.01	126	29.5 (26.4)	36.20	0.0061	0.72
**Total Cholesterol**	30690	6200	1.00	2	-	-	-	-
**LDL Cholesterol**	30780	6200	1.00	154	−22.3 (30.8)	41.80	0.13	<0.001
**HbA1c**	30750	5290	1.03	27284	−1.02 (2.04)	2.80	0.035	0.06
**Triglycerides**	30870	6211	1.01	136	−9.06 (25.2)	34.10	0.063	0.001
**C-reactive protein**	30710	6203	1.01	20268	−1.44 (1.88)	2.53	0.035	0.05

## Data Availability

The datasets generated and/or analysed during the current study are not publicly available due the Data Materials and Distribution Agreement that governs data use for the Jackson Heart Study and precludes the author(s) from sharing data but are available from the corresponding author on reasonable request.

## Abbreviations

CVD: Cardiovascular disease
CHD: Coronary heart disease
PRS: Polygenic risk score
UK: United Kingdom
GWAS: Genome-wide association study
JHS: Jackson Heart Study
IRB: Institutional review board
ASCVD: Atherosclerotic cardiovascular disease
SBP: Systolic blood pressure
DBP: Diastolic blood pressure
HDL: High-density lipoprotein
HbA1c: Hemoglobin A1c
CRP: C-reactive protein
SNP: single-nucleotide polymorphism
HWE: Hardy-Weinberg Equilibrium
MAF: Minor allele frequency
UKBB: United Kingdom Biobank
ACC/AHA: American College of Cardiology/American Heart Association
